# Supplementary material for: Optogenetic control of cell signaling pathway through scattering skull using wavefront shaping
Source: Sci Rep. 2015 Aug 21;5:13289. doi: 10.1038/srep13289 (PMC4543936; doi:10.1038/srep13289)
Supplement: Supplementary Information [file srep13289-s1.pdf]

Supplementary information for

Optogenetic control of cell signaling pathway through scattering skull using wavefront shaping

Jonghee Yoon[1,†], Minji Lee[2, †], KyeoReh Lee[1], Hyeonseung Yu[1], Nury Kim[2], Jin Man Kim[3], Jongchan Park[1], Chulhee Choi[4], Won Do Heo[2,5,*] and YongKeun Park[1,*]

[1]Department of Physics, Korea Advanced Institute of Science and Technology, Daejeon 305-701, Republic of Korea

[2] Department of Biological Sciences, Korea Advanced Institute of Science and Technology, Daejeon 305-701, Republic of Korea

[3]Graduate School of Medical Science and Engineering, Korea Advanced Institute of Science and Technology, Daejeon 305-701, Republic of Korea

[4]Department of Bio and Brain Engineering, Korea Advanced Institute of Science and Technology, Daejeon 305-701, Republic of Korea

[5]Center for Cognition and Sociality, Institute for Basic Science (IBS), Daejeon 305-811, Republic of Korea

[†]These authors contributed equally to this work.

Correspondence should be addressed to Y.-K.P (yk.park@kaist.ac.kr) or W.D.H (wondo@kaist.ac.kr)

Correspondence:

YongKeun Park, PhD

KAIST, 291 Daehak-ro, Yuseong-gu, Daejeon 305-701, Korea

Phone: +82-42-350-2514; Fax: +82-42-350-2510

Email: yk.park@kaist.ac.kr

Won Do Heo, PhD

KAIST, 291 Daehak-ro, Yuseong-gu, Daejeon 305-701, Korea

Phone: +82-42-350-2642; Fax: +82-42-350-2610

Email: wondo@kaist.ac.kr

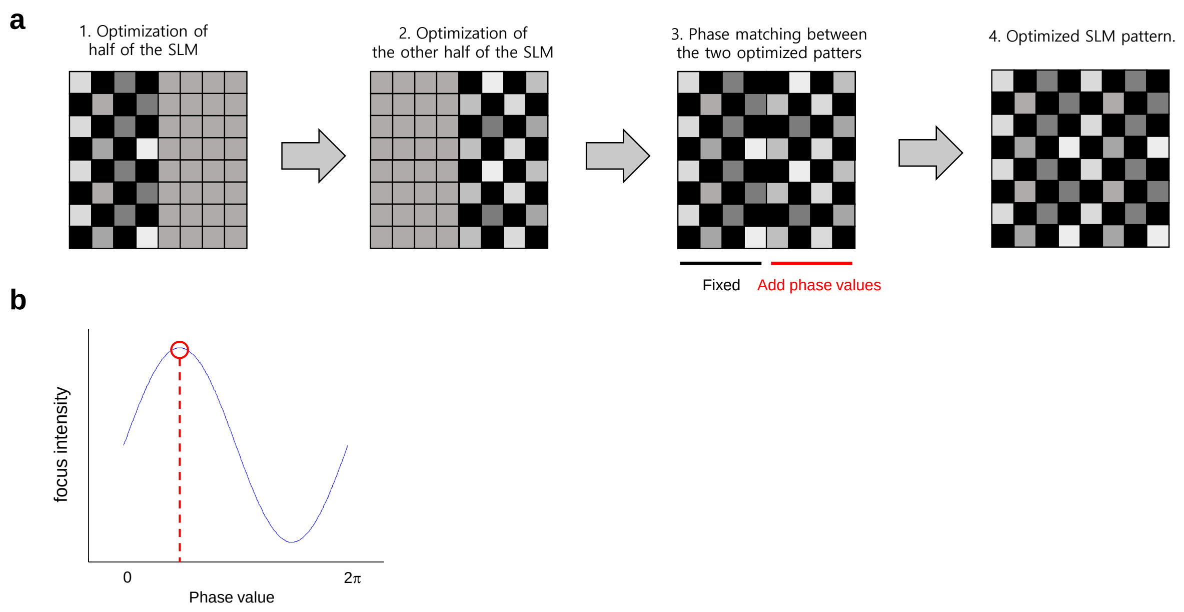

Supplementary Figure 1. Optimization procedure to find the optimized SLM pattern for focusing through the mouse skull: (a) Each half of the segment is optimized separately, and then the relative phase between each half of the segments is matched to achieve the optimized SLM pattern. (b) The intensity measured at the focus during the phase-matching process. The red circle indicates the maximum focus intensity.

Position	Mouse stain	Age (weeks)	Transport mean free path (mm)
Frontal bone	B6	6	0.204
	B6	20	0.475
	Balb/c	37	0.456
	C3H/HeJ	98	0.213
Parietal bone	B6	6	0.128
	B6	20	0.169
	Balb/c	37	0.116
	C3H/HeJ	98	0.179

Supplementary Table 1. Measured transport mean free paths of the frontal and parietal bones according to mouse strains and ages.
